# Supplementary material for: Ethanol facilitates socially evoked memory recall in mice by recruiting pain-sensitive anterior cingulate cortical neurons
Source: Nat Commun. 2018 Aug 30;9:3526. doi: 10.1038/s41467-018-05894-y (PMC6117351; doi:10.1038/s41467-018-05894-y)
Supplement: Supplementary file 1 — Supplementary Information [file 41467_2018_5894_MOESM1_ESM.pdf]

Supplementary Information

Ethanol facilitates socially evoked memory recall in mice by recruiting pain-sensitive anterior cingulate cortical neurons

Sakaguchi et al.

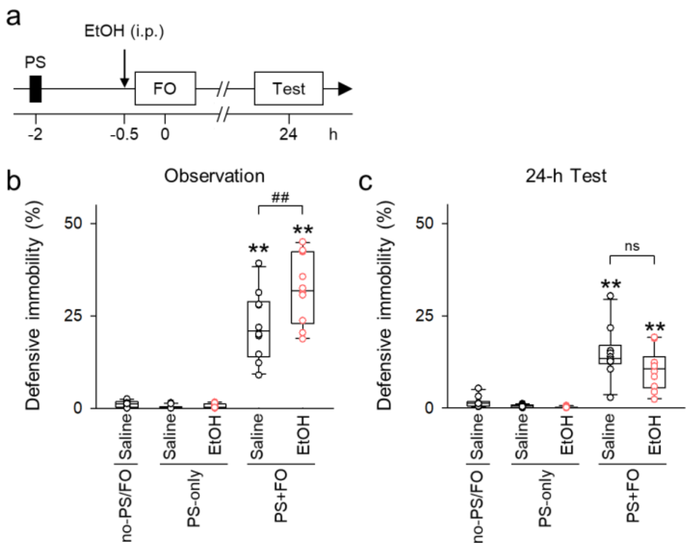

Supplementary Figure 1 | Mice learn fear-associated contexts by observing the behavior of a cagemate. (**a**) Experimental paradigm for examining whether the observers exhibit conditioned fear responses 24 h after the fear observation (FO) following a priming shock (PS). (**b,c**) The total immobility time of observers during the FO (**b**) and during the test 24 h after the observation (**c**). Box and whisker plots indicate medians (line with in box), first and third quartiles (bounds of box), and the distributions of 10% and 90% (whiskers). Systemic injection of 1.5 g/kg ethanol 30 min before the FO significantly increased immobility time during the FO but not during the test. $^{**}P < 0.01$ *versus* the no-PS/FO group with saline injection, $^{\#\#}P < 0.01$ *versus* the PS+FO group with saline injection, *t*-based bootstrap test after Kruskal-Wallis test, n = 10 mice per group.

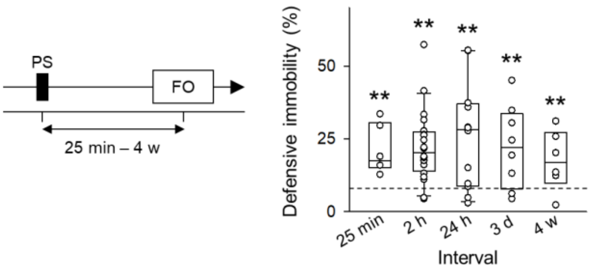

Supplementary Figure 2 | The facilitatory effect of a priming shock on observational fear lasts for at least 4 w. *Left*, Experimental paradigm for examining how long a single priming foot shock (PS) continued to enhance fear observation (FO). A priming foot shock was delivered 25 min, 2 h, 24 h, 3 d, or 4 w before the fear observation test. *Right*, PS-induced increases in defensive immobility times were consistently observed in all experimental groups. **$P < 0.01$ *versus* the average value of the FO-only group in Fig. 1b, which is indicated by the broken line. There was no significant difference across groups. $F_{4,47} = 0.390$, $P = 0.815$, one-way ANOVA, $n = 6$–20 mice per group.

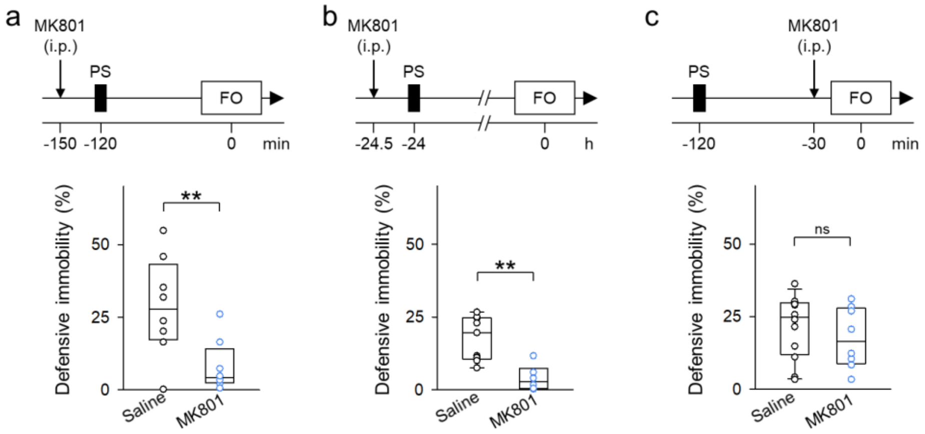

Supplementary Figure 3 | The facilitatory effect of a priming shock requires NMDA receptor activity. (**a–c**) *Top*, experimental paradigm for examining the involvement of NMDA receptor activity in the priming effect. MK801 (0.1 mg/kg), an NMDA receptor antagonist, was intraperitoneally injected 30 min before the priming shock (PS; **a,b**) or 30 min before the fear observation (FO; **c**). *Bottom,* Priming shock-facilitated observational fear response was abolished when MK801 was administered before the priming shock (**a**: $t_{14} = 3.00$, $^{**}P = 1.57\times10^{-4}$, t-based bootstrap test, $n = 8$ mice per group, **b**: $t_{13} = 4.43$, $^{**}P < 1.0\times10^{-4}$, $n = 6–9$ mice per group) but not before the fear observation test (**c**: $t_{18} = 0.812$, $P = 0.132$, $n = 8–12$ mice per group). Thus, the facilitatory effect forms through NMDA receptor-dependent plasticity during the priming shock.

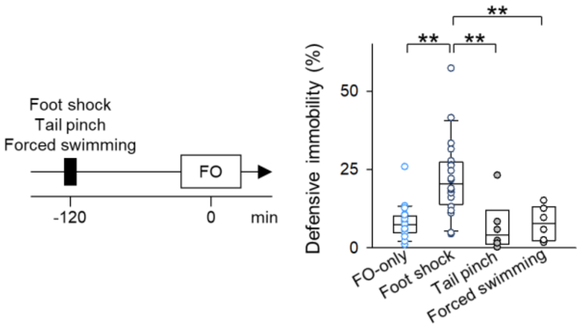

Supplementary Figure 4 | Neither tail pinch nor forced swimming affects subsequent fear observation. *Left,* Experimental paradigm for examining whether irrelevant pain (tail pinch) or stress (forced swimming for 15 min) facilitated observational fear response. *Right,* Mice that had undergone a tail pinch or forced swimming 120 min before the fear observation test exhibited defensive immobility times similar to the control level expressed by the FO-only mice. $^{**}P < 0.01$, *t*-based bootstrap test, $\chi^2_{3,48} = 21.5$, $P = 8.24\times10^{-5}$; Kruskal-Wallis test, $n = 6$–20 mice per group. Data from the FO-only and foot shock groups are the same as in Fig. 1b.

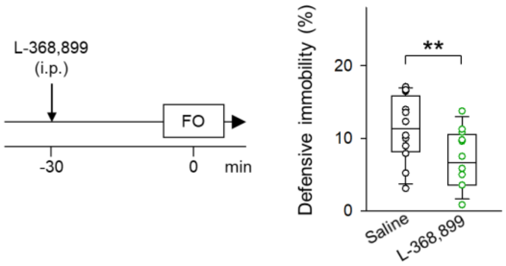

Supplementary Figure 5 | An oxytocin receptor antagonist suppresses fear transmission. *Left*, Experimental paradigm for examining the effect of 5 mg/kg L-368,899, an oxytocin receptor antagonist, on fear transmission. L-368,899 was intraperitoneally injected 30 min before the fear observation (FO). *Right*, L-368,899 decreased defensive immobility during the FO. $t_{22} = 2.43$, $^{**}P = 1.01 \times 10^{-3}$; t-based bootstrap test, $n = 12$ mice per group.

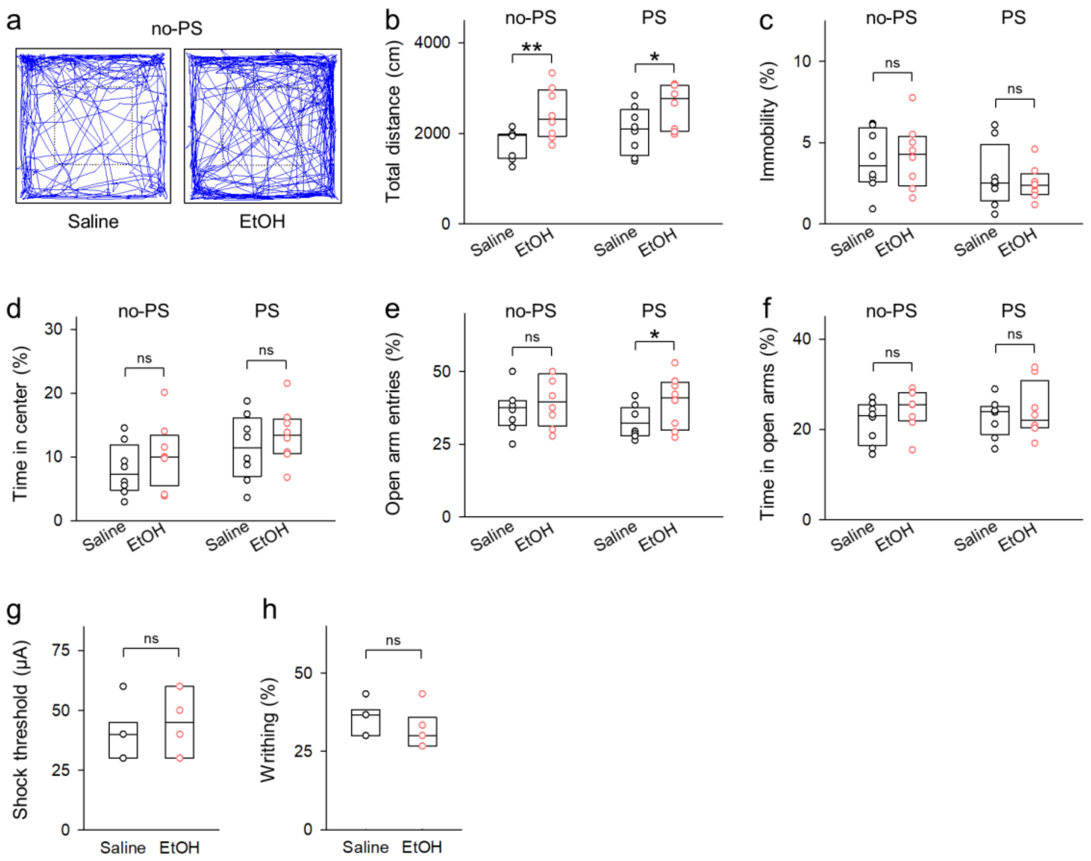

Supplementary Figure 6 | Ethanol does not reduce locomotor activity or increase pain sensitivity. (**a**) Representative traces of spatial movements of mice in an open square field for 10 min. (**b–d**) Ethanol at a dose of 1.5 g/kg increased the total distance travelled (**b**, **P* < 0.05, ***P* < 0.01, *t*-based bootstrap test after Kruskal-Wallis test, *n* = 8 mice per group) but did not affect the immobility time (**c**, $F_{3,28} = 1.61$, $P = 0.209$, one-way ANOVA) or the total time spent in the center area (**d**, $F_{3,28} = 1.81$, $P = 0.168$, one-way ANOVA). These results were not affected by a priming shock (PS) 2 h before the open field test. (**e,f**) Percentages of open-arm entries (**e**) and time spent on the open arms (**f**) in an elevated plus-maze test. Ethanol increased open-arm entries only after the PS (**e**, **P* < 0.05, *t*-based bootstrap test after one-way ANOVA, *n* = 8 mice per group), but it did not alter time spent in open arms (**f**, $F_{3,28} = 0.629$, $P = 0.602$, one-way ANOVA). Thus, ethanol induced behavioral hyperactivity and tended to reduce anxiety. (**g**) Ethanol did not modify the minimal intensity of electrical shocks needed to induce pain-related reflex muscle responses and vocalization. $U_{6,6} = 14.5$, $P = 0.350$, Mann-Whitney *U* test, *n* = 6 mice per group. (**h**) Ethanol did not change the frequency of

acetic acid-induced writhing responses. The frequency was measured as the mean probability (%) that writhing occurred during any given 5-s period of the 10-min video. Ethanol and acetic acid (0.9%) were intraperitoneally injected 30 and 5 min before the behavioral test, respectively. $U_{6,6} = 9.50$, $P = 0.120$, Mann-Whitney U test, $n = 6$ mice per group.

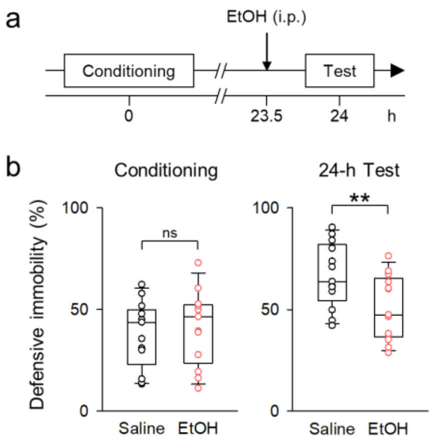

Supplementary Figure 7 | Ethanol suppresses contextual fear memory retrieval. (**a**) Experimental paradigm for examining the effects of 1.5 g/kg ethanol on memory recall in a classical fear conditioning paradigm. (**b**) Percentages of immobility time during conditioning (left) and 24-h test (right). Ethanol impaired fear memory retrieval. Conditioning: t_{24} = 0.474, P = 0.255; 24-h Test: t_{24} = 2.54, $^{**}P = 5.87\times10^{-4}$, t-based bootstrap test, n = 13 mice per group.

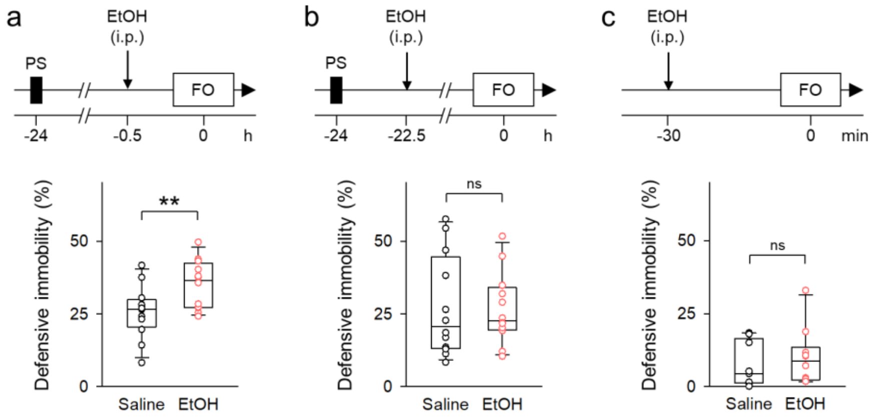

Supplementary Figure 8 | Ethanol does not enhance fear transmission in mice without a priming shock. (**a,b**) Ethanol enhanced observational fear esponse in mice that had experienced a priming shock when administered 30 min before the fear observation test (**a**, t_{22} = 2.62, **P = 4.80×10^{-4}, t-based bootstrap test, n = 12 mice per group) but not when administered 90 min after the priming shock (**b**, t_{22} = 8.70×10^{-2}, P = 0.454, n = 12 mice per group). (**c**) Ethanol did not affect fear transmission without a priming shock. t_{17} = 0.705, P = 0.162, n = 9–10 mice per group.

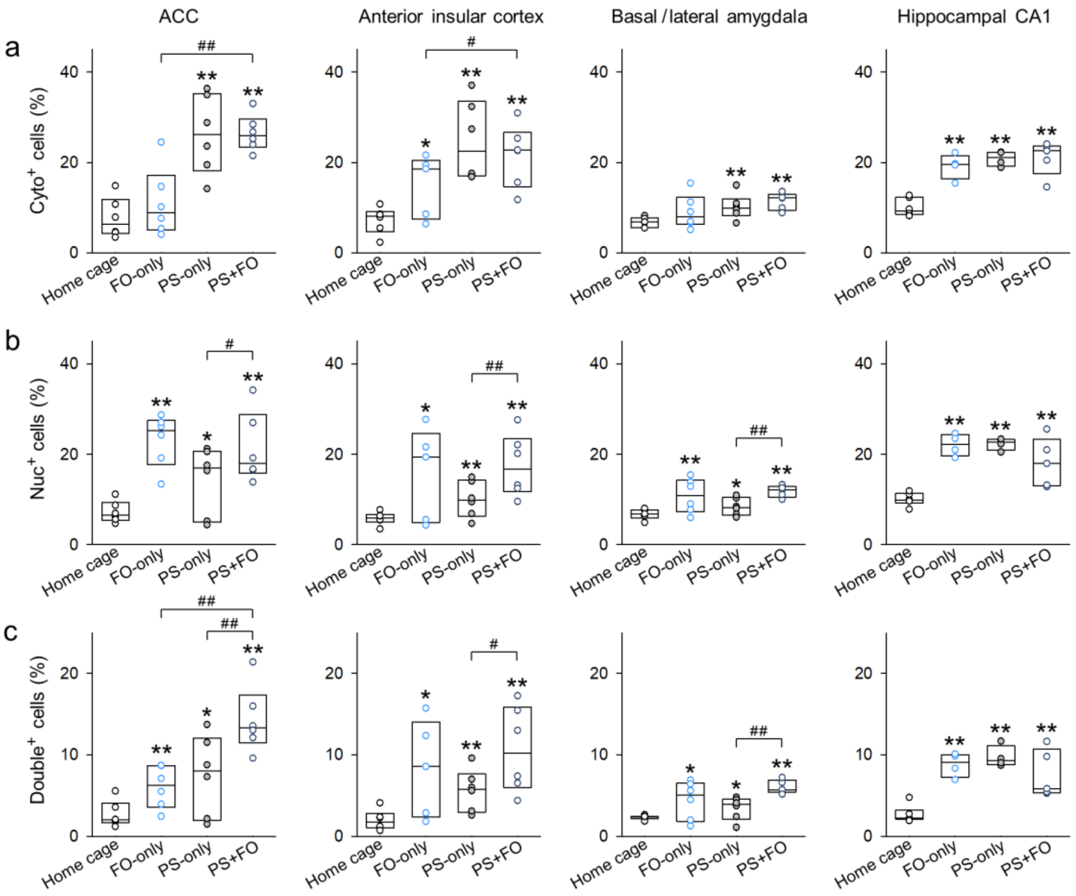

Supplementary Figure 9 | Temporal activity mapping with cellular resolution using *Arc* catFISH. (**a**–**c**) Percentages of cells with *Arc* expression in the cytoplasm (**a**), the nuclei (**b**), or both (**c**) relative to Hoechst-stained cells in the anterior cingulate cortex (ACC), the anterior insular cortex, the basal/lateral amygdala, and the dorsal hippocampal CA1 area. Data of Nuc^+ cells (**b**) from the ACC are the same as in Fig. 2c. $*P < 0.05$, $**P < 0.01$ *versus* home-cage group, $^{\#}P < 0.05$, $^{\#\#}P < 0.01$ *versus* the PS+FO group, *t*-based bootstrap test after one-way ANOVA, $n = 5–6$ mice per group.

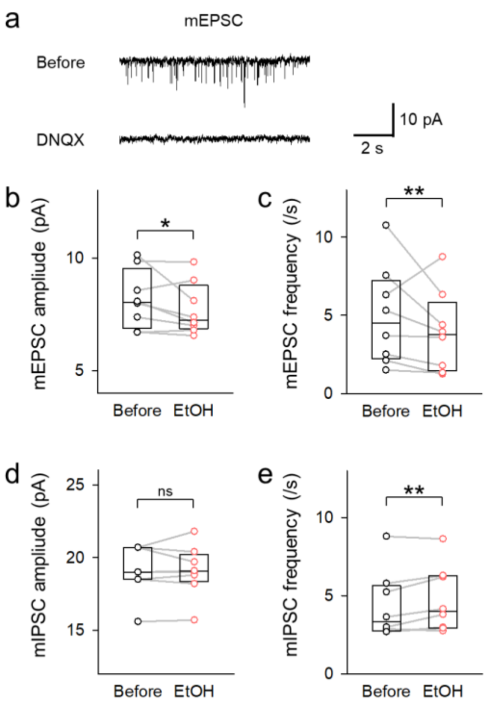

Supplementary Figure 10 | Ethanol shifts synaptic E/I balance toward inhibition in the ACC. (**a**) Representative whole-cell traces of mEPSCs in an ACC layer II/III pyramidal cell in an acute brain slice before and after bath application of 500 µM DNQX, an AMPAR antagonist. (**b–e**) Effects of ethanol (50 mM) on the amplitudes (**b,d**) and the frequencies (**c,e**) of mEPSCs and mIPSCs. Ethanol decreased the amplitudes and the frequencies of mEPSCs and increased the frequencies of mIPSCs. mEPSC amplitude: t_7 = 1.78, *P = 1.81×10^{-2}; mEPSC frequency: t_7 = 2.37, **P = 4.81×10^{-3}; mIPSC amplitude: t_7 = 1.17×10^{-2}, P = 0.495; mIPSC frequency: t_7 = 2.82, **P = 1.84×10^{-3}; t-based bootstrap test, n = 8 cells.

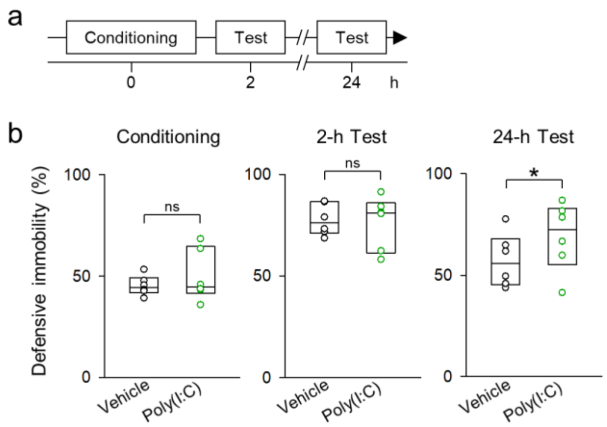

Supplementary Figure 11 | Poly(I:C) mice exhibit enhanced fear memory recall 24 h but not 2 h after the conditioning. (**a**) Experimental paradigm of contextual fear conditioning. (**b**) Percentages of defensive immobility of vehicle-treated and poly(I:C)-treated groups during the conditioning (*left*: t_{10} = 0.850, P = 0.135, *t*-based bootstrap test, n = 6 mice per group), the first test 2 h after the conditioning (*middle*: t_{10} = 0.219, P = 0.383), and the second test 24 h after the conditioning (*right*: t_{10} = 1.36, *P = 3.92×10^{-2}).

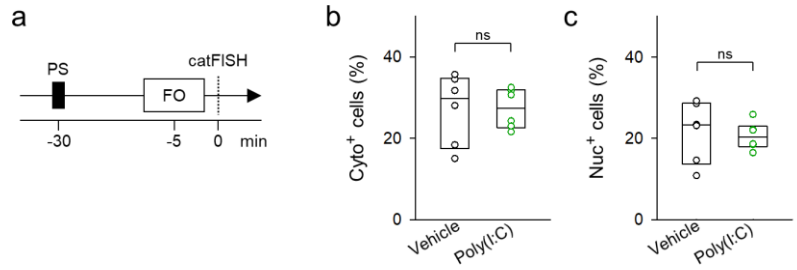

Supplementary Figure 12 | The numbers of cells that were activated during priming shock (Cyto$^+$) and those activated during observation (Nuc$^+$) do not differ between control and poly(I:C) mice. (**a**) Experimental paradigm for *Arc* catFISH. (**b,c**) Percentages of cells that expressed *Arc* mRNA in the cytoplasm (**b**, $t_{10} = 1.85\times10^{-2}$, $P = 0.490$, *t*-based bootstrap test, $n = 6$ mice per group) and the nuclei (**c**, $t_{10} = 0.305$, $P = 0.345$) in Hoechst-positive ACC cells were similar between control and poly(I:C) mice. Therefore, the reduced overlap scores in poly(I:C) mice cannot be explained by the activity level of ACC neurons.
